# Cutaneous Depigmentation in a Child With Ocular Albinism Type 1: Expanding the Clinical Phenotype

**DOI:** 10.7759/cureus.97178

**Published:** 2025-11-18

**Authors:** Matilde Monteiro, Joana Fazendeiro Matos, Joana Sá, Maria João Nabais Sá, Catarina Queirós

**Affiliations:** 1 Dermatology, Unidade Local de Saúde de Gaia/Espinho, Vila Nova de Gaia, PRT; 2 Center for Predictive and Preventive Genetics (Institute for Molecular and Cell Biology) i3S (Institute for Research and Innovation in Health), University of Porto, Porto, PRT

**Keywords:** genetic testing, gpr143, hypopigmentation, melanocytes, ocular albinism

## Abstract

Ocular albinism type 1 (OA1) is an X-linked disorder caused by mutations in the *GPR143* gene, leading to ocular features such as nystagmus, foveal hypoplasia, and reduced visual acuity. While GPR143 is involved in melanocyte function, clinically evident skin involvement is rarely reported. We describe a nine-year-old male with OA who presented with extensive, sharply demarcated depigmented patches, stable for over three years, and a confirmed pathogenic *GPR143* variant. This case expands the known phenotype of OA1 and highlights the need for further research into the cutaneous effects of GPR143 dysfunction.

## Introduction

Albinism encompasses a group of rare genetic disorders characterized by cutaneous and ocular hypopigmentation [1]. There are two main subtypes of albinism: oculocutaneous albinism (OCA), which affects the skin, hair, and eyes, and ocular albinism (OA), which primarily involves the eyes. These disorders are further subclassified based on the specific genetic variants involved. To date, seven genes have been associated with OCA (OCA1-7) [2].

OA type 1 (OA1), also known as Nettleship-Falls type, is the most common form of OA, with an estimated prevalence of approximately one in 50,000 individuals. It is inherited in an X-linked manner, thus occurring almost exclusively in males [2,3]. 

OA1 is caused by disease-associated variants in the *GPR143* gene, located on chromosome Xp22.2, which encodes a G protein-coupled receptor predominantly expressed in melanocytes and the retinal pigment epithelium [4]. This receptor plays a crucial role in the development and maturation of melanosomes [5], the intracellular organelles responsible for melanin storage [6]. Tissue pigmentation is determined by both the number and density of melanosomes, as well as the quantity of melanin deposited within them [7]. In the absence of functional GPR143 protein, melanosomes fail to properly bud from the endoplasmic reticulum and form giant structures known as macromelanosomes [3,5], detectable via light and electron microscopy in both retinal pigment epithelium and skin samples from OA1 patients [8]. Despite these structural abnormalities, the melanin synthesis machinery appears to remain intact in OA1 patients [9,10]. Consequently, the precise pathogenic mechanisms underlying the phenotype of disease-associated *GPR143* variants remain unclear [11]. 

Affected males with OA1 typically present with impaired visual acuity, nystagmus, strabismus, and photophobia. Ophthalmologic findings often include iris translucency, foveal hypoplasia, and hypopigmentation of the retina [2]. Female carriers are usually asymptomatic but may exhibit subtle findings such as iris transillumination defects, a characteristic "mud-splattered" appearance of the fundus, and foveal hypoplasia [1,12].

According to the current literature, cutaneous manifestations in OA1 are generally absent or mild. Most reports describe patients with OA1 as having normal skin and hair pigmentation [3,11,13,14]. Earlier studies noted that mild cutaneous hypopigmentation, when present, could only be detected through direct comparison with unaffected siblings [2]. More recently, López-Fontanet *et al.* documented a 22-year-old male with molecularly confirmed OA1 who exhibited well-defined depigmented patches on the elbows and thigh [15]. Thus, despite the central role of *GPR143* in melanocyte biology, clinically significant cutaneous involvement in OA1 remains rare and has only been sporadically reported.

We present the case of a nine-year-old male patient diagnosed with OA1 who presented with multiple well-demarcated depigmented cutaneous lesions. This report expands the phenotypic spectrum associated with OA1 and highlights the need for careful dermatological evaluation combined with genetic testing to ensure accurate diagnosis and appropriate management in these patients.

## Case presentation

We report the case of a nine-year-old male with a clinical diagnosis of OA, who presented with horizontal nystagmus and reduced visual acuity since early infancy. He was referred to the Dermatology Department for evaluation of cutaneous lesions first noticed around the age of six, which had remained stable during this period, with no new lesions appearing.

**Figure 1:**
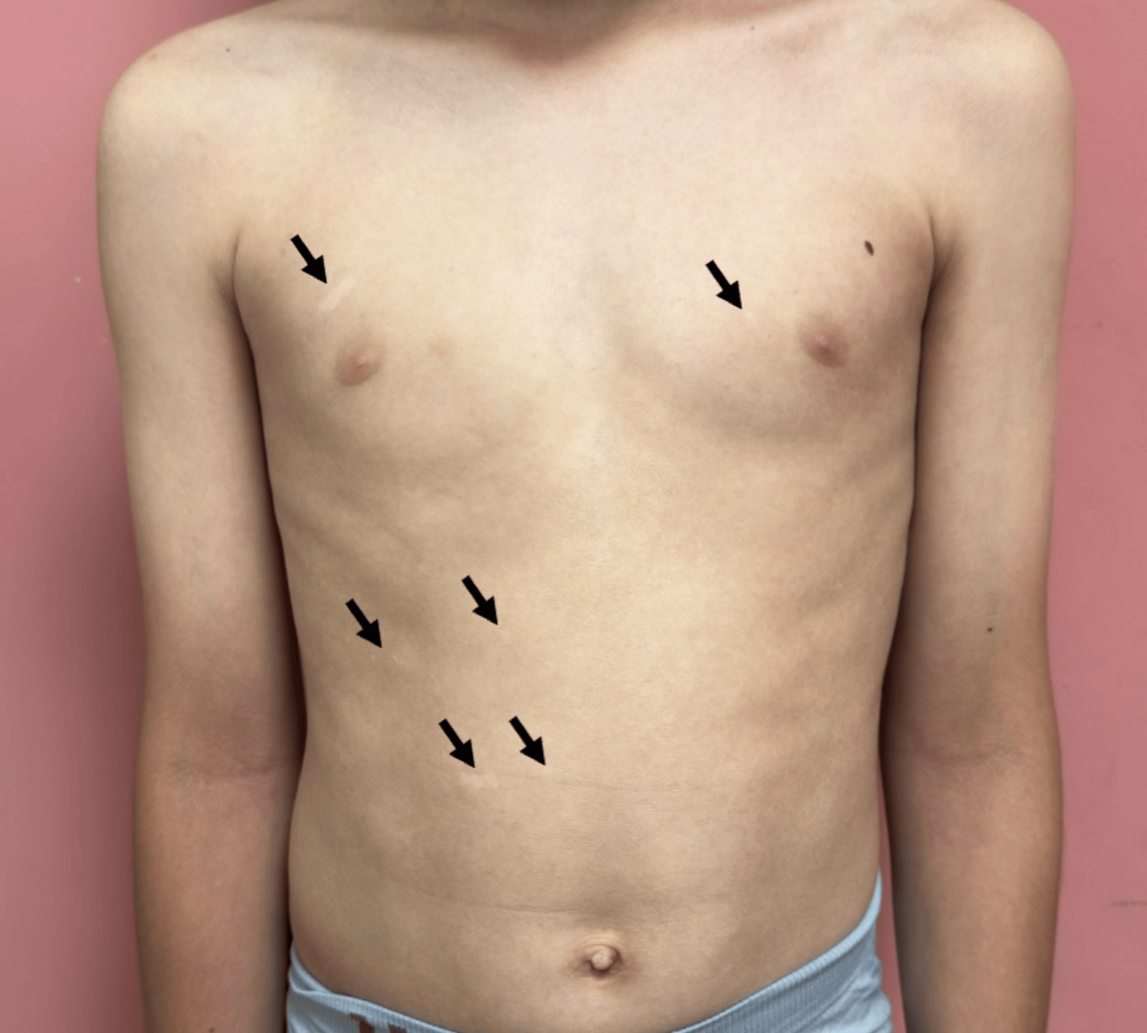
Multiple well-demarcated depigmented macules distributed across the anterior trunk (black arrows).

**Figure 2:**
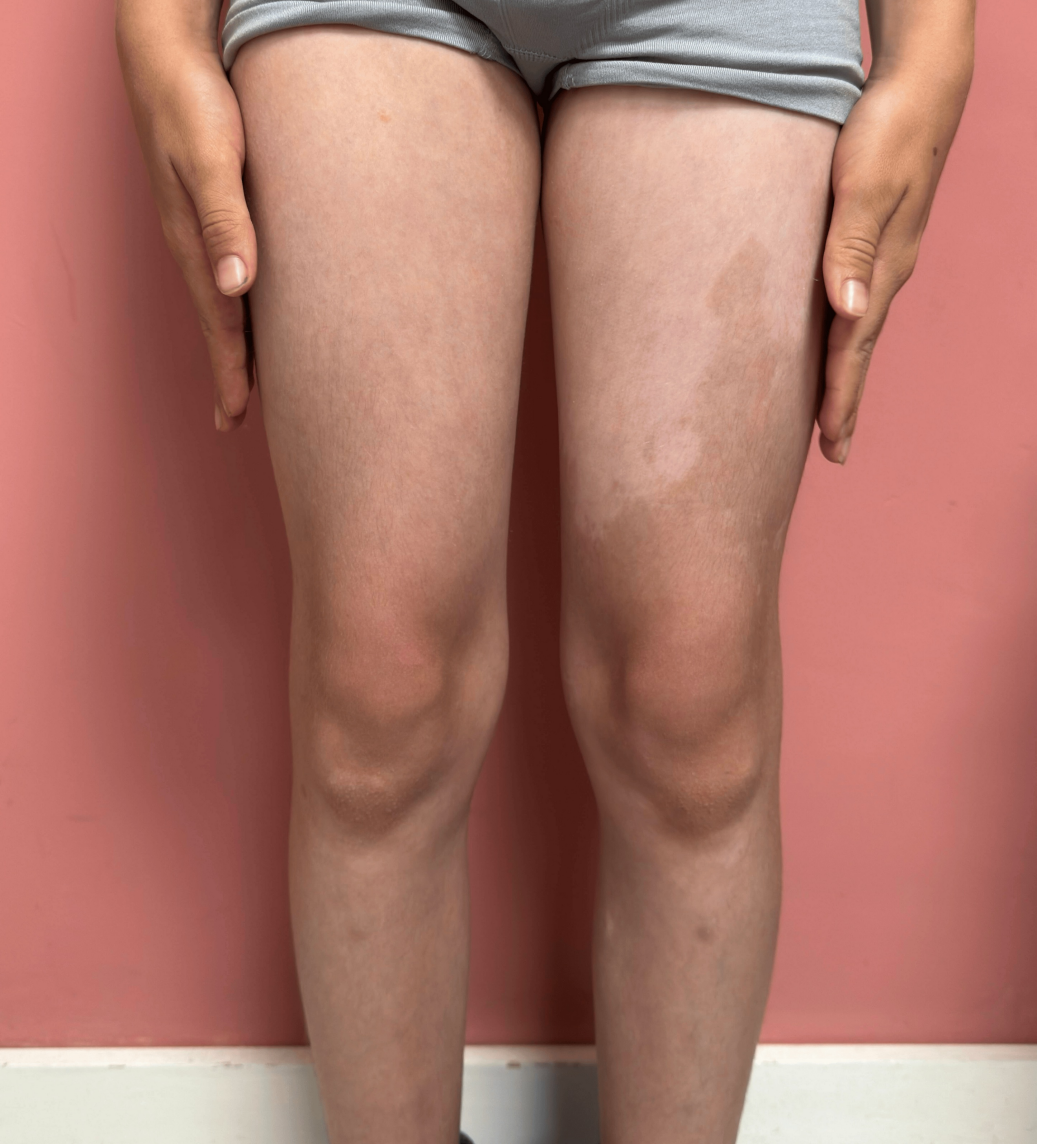
A large, depigmented patch involving nearly the entire anterior aspect of the left thigh. In direct comparison, a hypopigmented area with poorly defined borders is visible on the right thigh. Scattered smaller depigmented macules are also observed on both legs.

**Figure 3:**
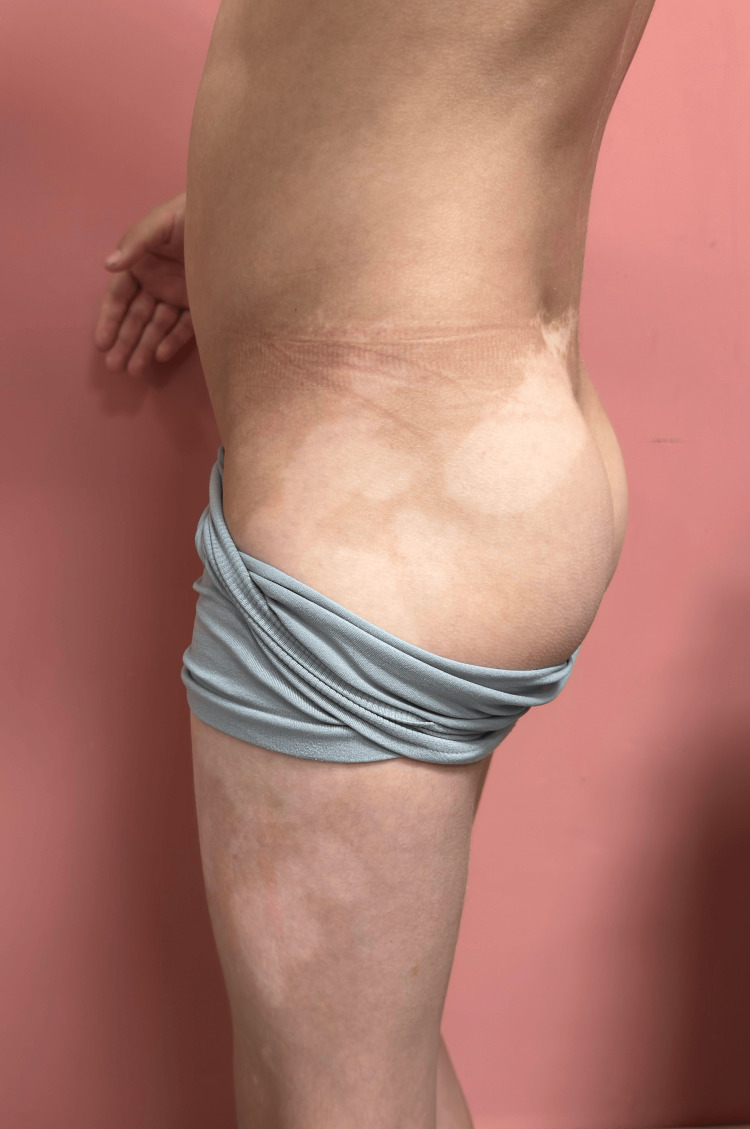
Continuation of the left thigh lesion along the lateral aspect of the left thigh, buttock and lumbar region, in the shape of multiple oval depigmented patches.

On dermatologic examination, multiple well-circumscribed depigmented macules and patches were observed. These lesions were distributed across the trunk, proximal extremities, and facial region (Figure 1). The most extensive lesion involved nearly the entire anterior and lateral aspects of the left thigh, with relative sparing of the posterior thigh (Figures 2-3). The right thigh also exhibited hypopigmentation with poorly defined margins. No signs of inflammation, scaling, or atrophy were present. Wood’s lamp examination confirmed the depigmented nature of the lesions, with an off-white accentuation. No *café-au-lait* macules or other pigmented skin abnormalities were noted.

Family history was notable for a maternal male cousin with similar hypopigmented skin lesions and a mother with documented visual impairment, raising the suspicion of X-linked inheritance. The patient’s younger sister exhibited no ocular or cutaneous manifestations.

**Figure 4:**
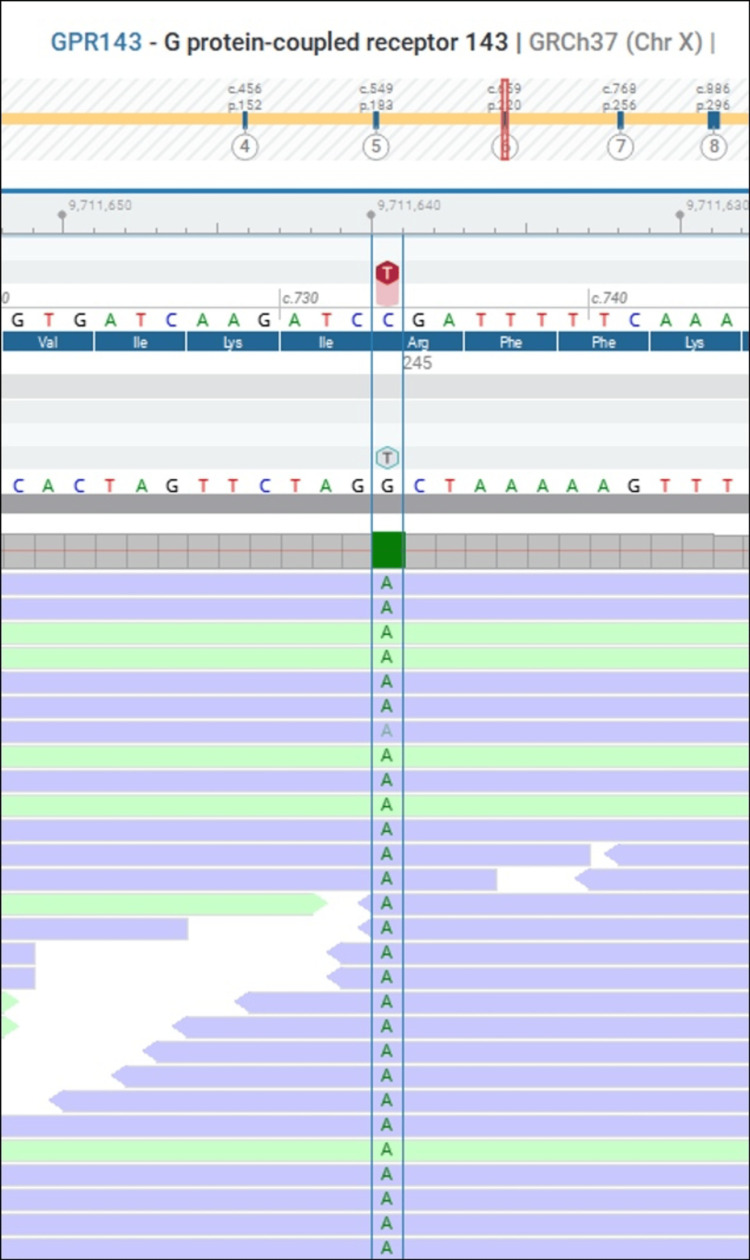
Analysis of a 27-gene panel for monogenic causes of oculocutaneous albinism, based on whole-exome sequencing, identified a pathogenic nonsense variant in hemizygosity in exon 6 of the GPR143 gene (c.733C>T; p.Arg245*). This variant introduces a premature stop codon and has been previously reported as causative of ocular albinism, consistent with an X-linked inheritance pattern.

A multigene panel for oculocutaneous albinism, based on whole-exome sequencing and comprising 27 genes, identified a hemizygous pathogenic nonsense variant in the *GPR143* gene (NM_000273.3:c.733C>T(p.(Arg245*), confirming the diagnosis of OA1 (Figure 4).

Given the patient’s age and the absence of diagnostic uncertainty following molecular confirmation, a skin biopsy was not performed to avoid unnecessary invasive procedures in a pediatric patient.

The patient and his immediate family members were referred to Medical Genetics for evaluation and counseling. Photoprotection measures were recommended, with particular emphasis on shielding the depigmented skin from ultraviolet radiation. At the six-month follow-up, the cutaneous lesions remained stable in distribution and appearance, and no new areas of depigmentation were identified. The patient continues to be monitored by both Dermatology and Ophthalmology Departments.

## Discussion

OA1 is an X-linked disorder characterized by ocular hypopigmentation and visual deficits, including nystagmus, reduced visual acuity, foveal hypoplasia, and iris translucency [2]. It is caused by variants in the *GPR143* gene, which encodes a protein crucial to melanosome development and function within melanocytes and the retinal pigment epithelium [5]. Despite the gene's role in melanocyte biology, cutaneous involvement in OA1 is classically minimal or absent. 

The current literature generally characterizes patients with OA1 as having normal skin and hair pigmentation [3,11,13,14], with only rare subtle skin hypopigmentation noted in earlier studies [2]. López-Fontanet *et al.* documented a 22-year-old male with OA1 who displayed well-defined depigmented patches on the posterior aspect of both elbows and the medial region of the right thigh, with histopathologic analysis excluding other differential diagnoses [15]. Here, we describe a child with OA1 who presented with extensive, well-demarcated depigmented skin lesions, including large patches on both thighs. This case further illustrates that significant skin involvement, although uncommon, can occur in this predominantly ocular condition, thereby expanding the known phenotypic spectrum of disease-associated *GPR143* variants.

These cutaneous manifestations may result from tissue-specific or amplified effects of *GPR143* variants. In most OA1 patients, skin melanocytes may compensate for GPR143 loss through redundant pathways, such as other GPCRs, Rab GTPases, or adaptor protein complexes [12]. In rare cases, however, regulatory differences may prevent this compensation, leading to visible skin involvement. Alternatively, variant-specific effects may also play a role; for example, truncated GPR143 proteins could accumulate in endosomal compartments, inducing endoplasmic reticulum or lysosomal stress and disrupting melanosome function.

The differential diagnosis included vitiligo and piebaldism, both of which were excluded on clinical and genetic grounds. Vitiligo, an acquired autoimmune condition, typically presents with progressive depigmentation with a predilection for areas around orifices, genitals, and hands, often with perifollicular hyperpigmentation, koebnerization, and a positive personal or family history of autoimmune diseases [16], all of which were absent in our patient. Piebaldism was unlikely given the lack of leukoderma since birth, absence of a white forelock, and absence of KIT or SLUG gene variants [17].

A skin biopsy was considered but ultimately not performed in order to avoid an invasive procedure, given the patient's age and the presence of a definitive genetic diagnosis. While histological analysis could show the presence of melanocytes, distinguishing from vitiligo, where melanocytes are absent [16], and macromelanosomes, the diagnostic utility was limited given the molecular genetic confirmation of the diagnosis.

## Conclusions

This case illustrates that, although rare, cutaneous involvement in OA1 may occur and should not exclude a clinical diagnosis. The presence of large, depigmented skin lesions in a patient with a confirmed *GPR143* mutation raises important questions about the phenotypic variability of OA1 and suggests that additional, yet unidentified, modifying factors may influence cutaneous pigmentation. Given that the melanin synthesis machinery appears to remain intact in OA1, further research is necessary to elucidate the underlying pathogenic mechanisms by which disease-associated *GPR143* variants may contribute to cutaneous pigmentary anomalies. A deeper understanding of these mechanisms may provide novel insights into melanosome biology and the broader spectrum of disorders affecting pigmentation.
